# COVID-19 Outbreaks in Settings With Precarious Housing Conditions in Germany: Challenges and Lessons Learned

**DOI:** 10.3389/fpubh.2021.708694

**Published:** 2021-09-21

**Authors:** Ruth Zimmermann, Navina Sarma, Doris Thieme-Thörel, Katharina Alpers, Tanja Artelt, Karima Azouagh, Viviane Bremer, Petra Broistedt, Tim Eckmanns, Nicolas Feltgen, Matthew Huska, Stefan Kröger, Angelika Puls, Simone Scheithauer, Eckart Mayr, Ute Rexroth

**Affiliations:** ^1^Department of Infectious Disease Epidemiology, Robert Koch Institute, Berlin, Germany; ^2^Local Public Health Authority, Göttingen, Germany; ^3^Infection Control and Infectious Diseases, University Medical Center Göttingen, Göttingen, Germany; ^4^Mayor's Office, Göttingen, Germany; ^5^Göttingen City Councillor, Göttingen, Germany; ^6^Department of Ophthalmology, University Medical Center Göttingen, Göttingen, Germany; ^7^Department of Methodology and Research Infrastructure, Robert Koch Institute, Berlin, Germany

**Keywords:** housing, overcrowding, poverty, marginalization, outbreak, SARS CoV-2, COVID-19

## Abstract

Two COVID-19 outbreaks occurred in residential buildings with overcrowded housing conditions in the city of Göttingen in Germany during May and June 2020, when COVID-19 infection incidences were low across the rest of the country, with a national incidence of 2.6/100,000 population. The outbreaks increased the local incidence in the city of Göttingen to 123.5/100,000 in June 2020. Many of the affected residents were living in precarious conditions and experienced language barriers. The outbreaks were characterized by high case numbers and attack rates among the residents, many asymptomatic cases, a comparatively young population, and substantial outbreak control measures implemented by local authorities. We analyzed national and local surveillance data, calculated age-, and gender-specific attack rates and performed whole genome sequencing analysis to describe the outbreak and characteristics of the infected population. The authorities' infection control measures included voluntary and compulsory testing of all residents and mass quarantine. Public health measures, such as the general closure of schools and a public space as well as the prohibition of team sports at local level, were also implemented in the district to limit the outbreaks locally. The outbreaks were under control by the end of June 2020. We describe the measures to contain the outbreaks, the challenges experienced and lessons learned. We discuss how public health measures can be planned and implemented through consideration of the needs and vulnerabilities of affected populations. In order to avoid coercive measures, barrier-free communication, with language translation when needed, and consideration of socio-economic circumstances of affected populations are crucial for controlling infectious disease transmission in an outbreak effectively and in a timely way.

## Introduction

The severe acute respiratory syndrome coronavirus 2 (SARS-CoV-2) is easily transmissible by both symptomatic and asymptomatic individuals. Closed and densely inhabited environments favor transmission (1–3).

The first COVID-19 case in the district of Göttingen was reported on March 11, 2020 (4). The whole district of Göttingen (city and county district) has a total population of ~330,000 (of whom ~120,000 live in the city), and is located in Lower Saxony, Germany. The district and the rest of Germany first reported an increase in incidence of COVID-19 during March with a peak in April—in Göttingen mainly due to outbreaks in care facilities—followed by a decrease in case numbers by the end of April 2020 (5). In May 2020, the first lockdown was released in whole Germany, and schools, shops and businesses re-opened and face masks were not recommended. In this situation, a COVID-19 case cluster was identified in a large residential complex (RC) in the city of Göttingen (RC1), followed by a second major outbreak in another RC (RC2) which was located 1 km away from RC1 but had no connections through residents. This led to increasing incidences in the whole district, which gained wide public attention (6–8).

We describe the two outbreaks in the RCs, with a particular focus on the housing and social conditions of the affected population and the challenges and measures taken to contain the outbreaks. We present further recommendations for the management of outbreaks that occur in buildings with cramped housing conditions, which often affect marginalized people who experience precarious living conditions.

## Materials and Methods

An outbreak investigation team from the Robert Koch Institute (RKI) consisting of two epidemiologists and four containment scouts supported the local health authorities from June 15–23, 2020, in implementing measures to understand and contain the outbreak.

The description of the two outbreaks was based on the COVID-19 database of the Local Public Health Authority of the City of Göttingen (LPHA), as of July 03, 2020.

Cases were defined as people who tested positive for SARS-CoV-2 by PCR between May 16 and June 29, 2020 [corresponding to calendar weeks (CW) 20–27, 2020], regardless of clinical symptoms, living in or with epidemiological link to either RC1 or RC2 in this period. The relevant information was gathered through the LPHA's investigations and entered in the local COVID-19 database. We described the COVID-19 cases in the whole district of Göttingen in two ways:

(a) All cases in the whole district of Göttingen from March 9 to August 9, 2020 (CW 11–32): by date of reporting, including cases assigned to the two outbreaks in RCs, based on data from the national surveillance database (RKI SurvNet, as of 13.08.2020).
(b) Cases assigned to outbreak 1 and 2 by date of testing, derived from the LPHA's COVID-19 database, differentiating between residents and non-residents and including information on local testing, screening and control measures in the two residential complexes, at Göttingen district and city level and at national level, May 16–June 29, 2020 (CW 20–27).

The age and gender distribution, and housing and social conditions of the residents of RC1 and RC2 were described based on information provided by the City of Göttingen, as well as the RKI's outbreak investigation team's personal impressions and their conversations with residents during their visit to the sites.

We compared gender and age distributions between cases among residents in RC1 and RC2 with the official total resident population, using the COVID-19 database of the LPHA and information provided by the City of Göttingen, and calculated age- and gender-specific attack rates (AR) among resident-cases.

Age, gender, and clinical symptoms were compared between outbreaks cases and national case numbers (by reporting date) during the outbreak period (May 16 to June 29, 2020; RKI SurvNet, as of 03.07.2020).

Whole genomes sequencing (WGS) was performed for 19 cases from the outbreak in RC2. The samples were sequenced on an Illumina iSeq using the Paragon Genomics CleanPlex SARS-CoV-2 Panel kit. The panel contains 344 primer with a median insert size of 96 base pairs (bp). The median sequencing depth was 100,000 reads per sample with a read length of 2x 125bp. Sequencing reads were used to reconstruct consensus genomes using CovPipe v2.0.1 (9). Minimum base quality was 15. Genome positions with a coverage of at least 20× were used for further analysis. Sequence similarity was calculated by using a multiple sequence alignment produced by Pangolin 2.3 (https://github.com/cov-lineages/pangolin/), which masks positions 0–265 and 29674-end of the sequences in reference coordinates. From the alignment, genetic distance was computed using a custom R script, ignoring positions with ambiguous nucleotides.

## Results

Between May 16 and June 29, 2020, the LPHA Göttingen identified 333 cases that were assigned to two outbreaks in two RCs located in the center of Göttingen. The first cluster comprised 71 residents in RC1 as well as 124 non-residents (outbreak 1), and the second cluster comprised 138 residents in RC2 (outbreak 2). No cases among non-residents were assigned to outbreak 2 by the LPHA.

RC1 consists of two buildings, one with 15 and another with 17 floors, and each with one staircase and two elevators. In total, there are 406 apartments, most of which are studios or have one bedroom (32–54 m^2^) and some 3–4 room apartments (71–78 m^2^). In May 2020, a total of 615 residents were officially registered; their gender and age distribution are shown in Tables 1, 2. The heterogeneous residential population consisted mostly of families and international students. According to the city authorities, more than half of the residents did not have a German passport (*n* = 331; 54%), and more than half received social benefits.

**Table 1:** Gender distribution of SARS-CoV-2 cases and officially registered residents of two residential complexes; gender-specific attack rates, Göttingen, May–June 2020 (COVID-19 database LPHA Göttingen, as of 03.07.2020).

	**Residential complex 1**					**Residential complex 2**				
	**Cases**		**Residents**		**Attack rate (%)**	**Cases**		**Residents**		**Attack rate (%)**
**Gender**	***n***	**%**	***n***	**%**		***n***	**%**	***n***	**%**	
Female	25	35.2	219	35.6	11.4	62	44.9	259	40.3	23.9
Male	46	64.8	396	64.4	11.6	76	55.1	384	59.7	19.8
**Total**	**71**	**100.0**	**615**	**100.0**	**11.5**	**138**	**100.0**	**643**	**100.0**	**21.5**

*The Totals are in bold values*.

**Table 2:** Age distribution of SARS-CoV-2 cases and officially registered residents of two residential complexes and age-specific attack rates, Göttingen May–June 2020 (COVID-19 database LPHA Göttingen, as of 03.07.2020).

	**Residential complex 1**					**Residential complex 2**				
**Age group**	**Cases**		**Residents**		**Attack rate [%]**	**Cases**		**Residents**		**Attack rate [%]**
	***n***	**%**	***n***	**%**		***n***	**%**	***n***	**%**	
0–5 years	11	15.5	39	6.3	28.2	21	15.2	89	13.8	23.6
6–17 years	15	21.1	41	6.7	36.6	42	30.4	122	19.0	34.4
18–44 years	35	49.3	363	59.0	9.6	56	40.6	290	45.1	19.3
45–64 years	8	11.3	131	21.3	6.1	19	13.8	121	18.8	15.7
65+ years	2	2.8	34	5.5	5.9	0		21	3.3	0
Unknown	0		7	1.1	0	0		0		0
**Total**	**71**	**100.0**	**615**	**100.0**	**11.5**	**138**	**100.0**	**643**	**100.0**	**21.5**

*The Totals are in bold values*.

RC2 is a dilapidated building with 432 apartments (17–39 m^2^), of which most are studios or have one bedroom; it extends over up to 12 floors, at that time with one functioning elevator for the entire building. Residents reported heavy littering of the building and availability of two washing machines for the residents, one of which was broken. In April 2020, 643 people were officially registered in the building, 60% of which were male and one third children under the age of 18. The gender and age distribution of residents are shown in Tables 1, 2. A total of 85% of the residents did not have German citizenship. According to local authorities, many residents had recently migrated to Germany. Of all residents in RC2, 90% (*n* = 578) received social benefits.

### Outbreaks 1 and 2 and Measures Taken

On May 18, 2020, a resident of RC1 developed clinical symptoms and tested positive for SARS-CoV-2. He was defined as the primary case. He did not comply with the isolation order. Another resident was hospitalized with pneumonia on May 25, 2020, and tested positive on May 26, 2020. Retrospectively, the LPHA identified three more persons linked to the primary case who tested SARS-CoV-2 positive on May 16, 17, and 20, respectively. Testing of 40 contacts of the identified four cases between May 26 and 29 revealed 38 more cases. Additional contact tracing of the new cases revealed a total of 364 close contacts, mostly non-residents of RC1.

Between May 30 and June 02, 2020, all 615 residents of RC1 were offered voluntary testing in various test centers. Sixty additional cases (residents and non-residents of RC1) were identified. Since not all residents attended the testing center, compulsory testing of the residents was conducted between June 05 and 07 in the garage of the building. Among 420 people tested, another 24 cases were identified (**Figure 3**).

Until June 12, 2020, the outbreak included 195 cases in total, of which 71 were residents of RC1.

On June 12, 2020, two residents from two households of RC2 tested positive for COVID-19 during outpatient consultation at the hospital. All five household contacts were tested positive. Thereafter, a screening of all residents was ordered for June 15 and 16, 2020. Among ~700 tested residents, 120 people tested positive for SARS-CoV-2. Retesting of negatives revealed 18 further cases, totalling 138 confirmed cases among residents of RC2 (**Figure 3**).

### Description of Cases by Time

**Figure 1:**
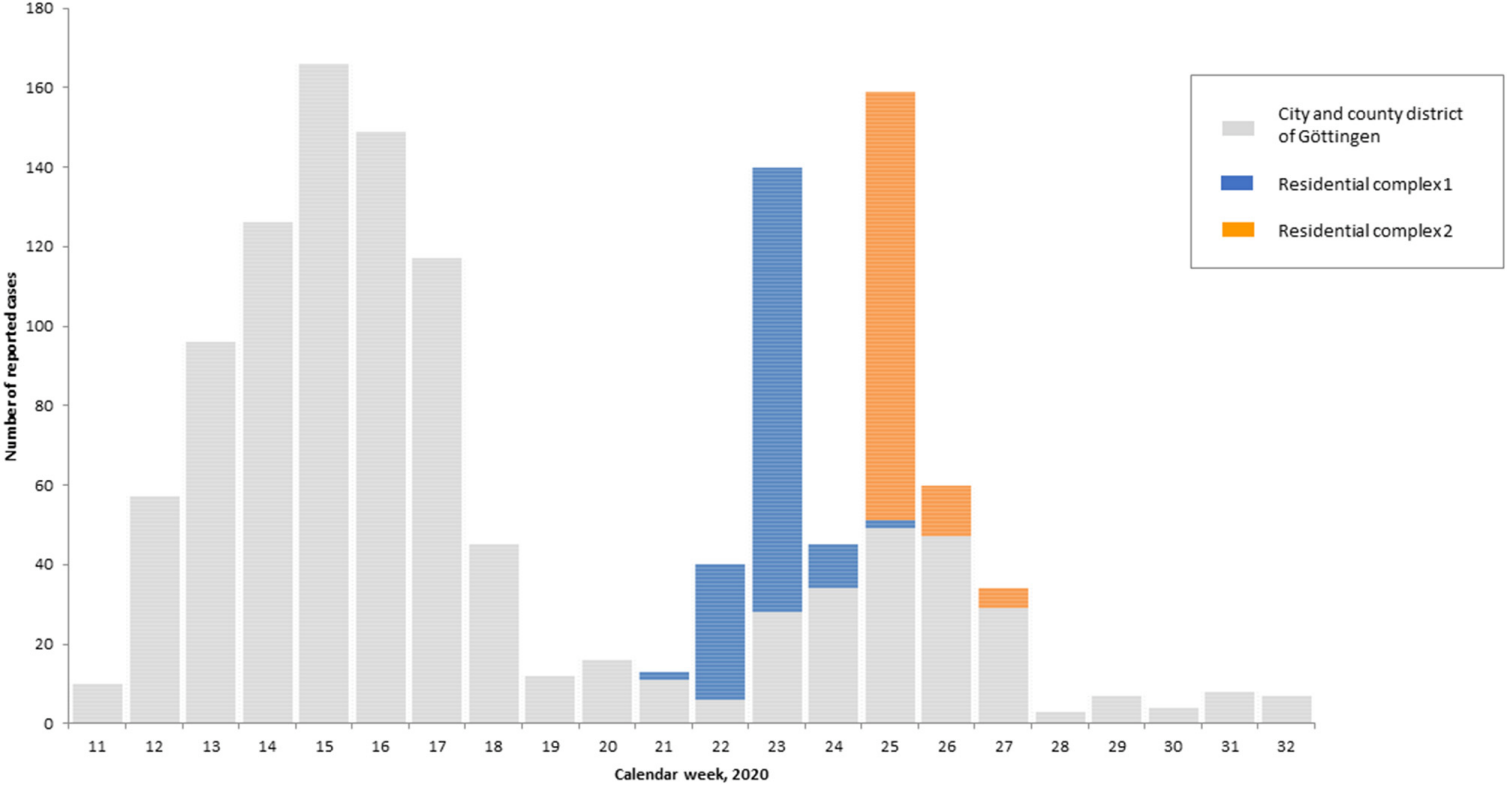
Distribution of SARS-CoV-2 cases (*N* = 1,323) reported to the RKI by reporting date in the calendar weeks 11–33, city and county district of Göttingen, 2020, with assignment of the cases to the outbreak events in residential complexes 1 (blue) and 2 (orange) (*n* = 287; RKI SurvNet database, 13.08.2020).

Figure 1 shows the reported COVID-19 cases in the whole district of Göttingen since the beginning of the epidemic. The cases assigned to the two outbreaks are highlighted. From May 18 to June 29 (CW 21–27), 58.5% (287/491) of cases in the whole district were attributed to the RC outbreaks. After an increase in case number in spring up to the peak of 166 cases in CW 15, case numbers decreased to 12–16 cases per week (CW 19–21). Following this, case numbers reached a similarly high level as in spring due to the outbreaks in RCs. In CW 23 and 25, *n* = 140 and 159 confirmed COVID-19 cases were reported, respectively; 80.0 and 69.2% of which could be attributed to the outbreak events (Figure 1).

### Gender and Age-Specific Attack Rates

In RC1, 71 of the 615 residents (and 124 non-residents) tested positive for SARS-CoV-2 (AR_RC1_ = 11.9%). In RC2, 138 of the 643 officially registered residents tested positive for SARS-CoV-2 (AR_RC2_ = 21.5%). No further cases outside the RC2 were identified. In RC2, the AR among women and men was 23.9 and 19.8%, respectively; in RC1 no gender difference of the AR was observed (Table 1).

The proportion of cases among children and adolescents under the age of 18 in RC1 and RC2 was 37 and 46% of all cases, respectively. In RC1, most cases (49%) occurred in the age group 18–44 years, in RC2 this age group contributed to 40% of cases. People aged over 45 years accounted for 14% share of cases in both RCs.

In both RCs, age-specific ARs were highest in children aged 6–18, followed by children aged 0–5, adults in age-group 18–44. The lowest ARs were among the oldest age-group (45–64) (Table 2).

### Whole Genome Sequencing Analysis of a Subset of Samples From Outbreak 2

**Figure 2:**
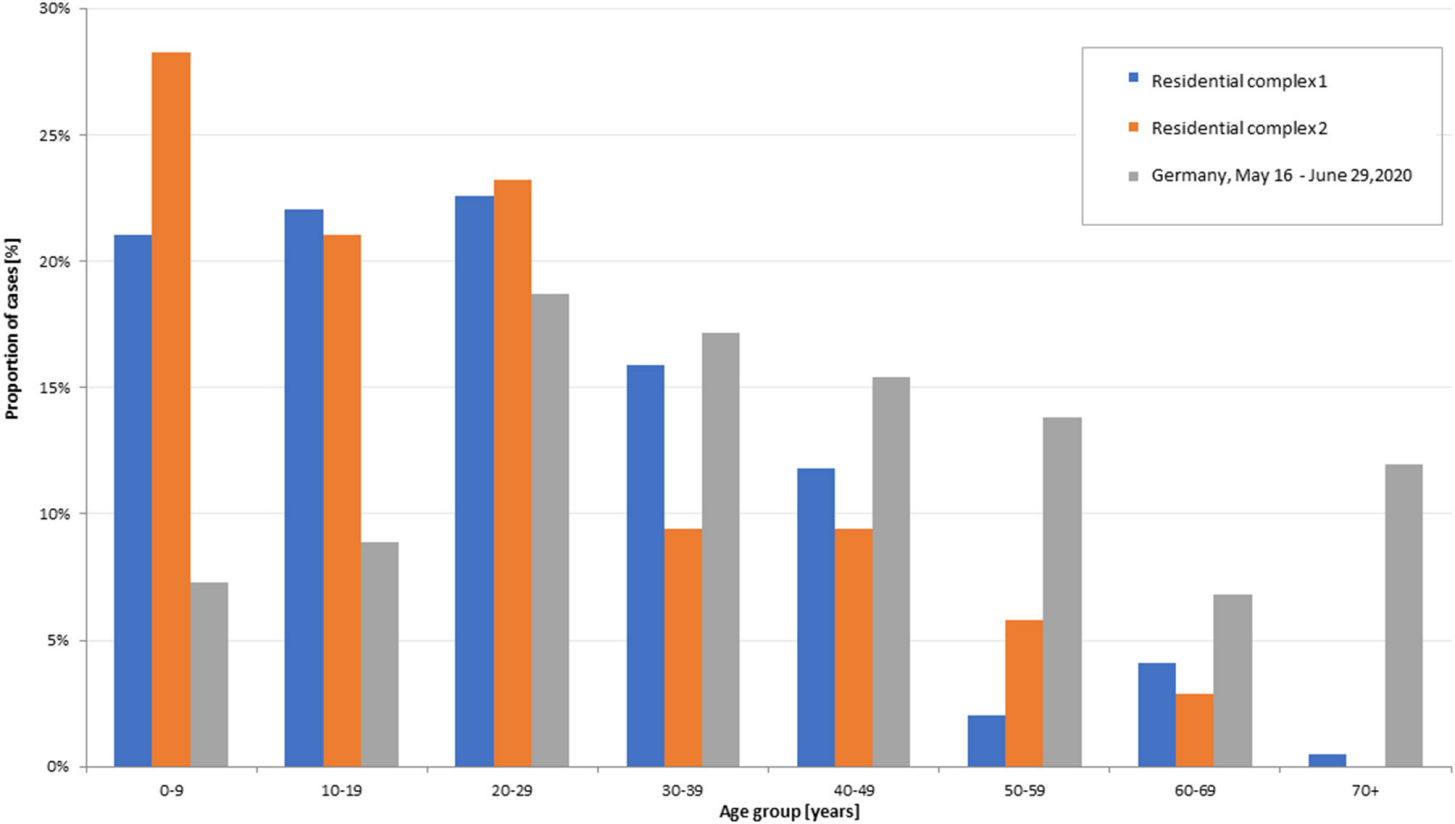
Age distribution of SARS-CoV-2 cases in outbreak 1; *n* = 195 and outbreak 2; *n* = 138 (COVID-19 database LPHA Göttingen, as of 03.07.2020), and in Germany (reporting week 20–27, SARS-CoV-2 cases according to reference case definition (*n* = 24,411; RKI SurvNet database, as of 03.07.2020).

WGS analysis of 19 samples from RC2 cases revealed high genomic sequence similarity with a maximal genomic distance between all of the samples between 0 and 3 SNPs (mean 1.1 SNPs) (Supplementry Figure 2).

There is no evidence of multiple introductions. Phylogenetic analysis revealed identical virus lineages B.1.159 in all samples. Despite relatively low overall lineage detection in Germany in 2020, B.1.159 was detected in only nine other samples in Germany. The 19 cases were spread over the whole RC2 with at least one sequenced case per level in one of the four building parts. The subset contained 11 children between the age of 0 to 15 and eight adults between the ages of 18–60. Based on age distribution in combination with locations of the flat, it can be excluded that these 19 cases define a separate subcluster within the RC2 or within outbreak 2 (Supplementry Figure 1).

### Age and Gender Distribution of Outbreak Cases in Comparison to the National Average

The overall proportion of women among all outbreak cases (including non-residents) was 45.6% (152/333), which was below the national average of 52.5% in the same period. In comparison to national COVID-19 cases in the same period, the proportion of outbreak cases was higher among children and adolescents, especially in outbreak 2. The cases' mean age was 25 years (median 23 years) in outbreak 1 and 23 years (median 20 years) in outbreak 2 compared to 48 years in the national average (median 49 years). Nearly 50% of the cases were aged under 20 years (in national case numbers this group accounted for <20% in the same time period). In contrast, the age groups over 40 years and older were underrepresented in the outbreaks compared to the nationally reported cases (Figure 2).

### Symptoms and Hospitalization

Information on clinical symptoms was available for a total of 150 cases, outbreak 1, in 135/195 (69.2%), and outbreak 2 in 15/138 (10.9%). Frequently cited symptoms among the cases with information on symptoms were fever (44.7%), cough (42.7%), headache (42.7%), sore throat (28%), and rhinorrhoea (21.3%). Ageusia and/or anosmia were indicated in 13 cases (8.6%), pneumonia in four cases (2.6%). Hospitalization was reported for 4.8% of cases [13 cases (6.7%) in RC1; and 3 cases (2.2%) in RC2]. Of the hospitalized cases, three people were treated in intensive care units (ICU) (2 <30 years, and >60 years). Two of them had to be ventilated, and one person died 26 days after diagnosis. The case fatality during the outbreak in outbreak 1 was 0.5% and 0 in outbreak 2.

### Measures to Contain the Outbreaks

On June 9, 2020, the Göttingen city crisis management team initiated a daily infection mapping based on a local map in order to early identify spatial signals of increased case numbers at crucial settings, such as nursing homes, hostels for refugees, and hostels for people who sleep rough, and precarious residential properties. To be able to quickly react to those signals, 50 medical students and trained staff from the university hospital (UMG) were available for mass testing and contact tracing.

#### Outbreak 1

In outbreak 1, testing of residents and contact persons, initially performed on a voluntary basis, was later made compulsory. Individual isolation was ordered for infected people and quarantine (for 14 days) for all members of households with newly identified cases. The residents independently organized the separation of cases, contacts and suspected negative-tested residents within the RC as well as food supply of quarantined residents (10). The primary case who did not comply with the isolation order was placed in a separate apartment. Appropriate ventilation and physical distancing among residents in the narrow corridors and elevators of the building were hardly possible. The city therefore asked the building's property management to submit an infection prevention and control (IPC) plan. It included regular cleaning, distancing, wearing of masks, and use of elevators by a maximum of two people at any one time.

Since two contact persons worked in two nursing homes for elderly people, all residents, and staff in the nursing homes were tested for SARS-CoV-2. One resident tested positive, no further cases occurred.

Thirty-three children who were infected had attended classes in reduced class sizes in 13 schools and 1 day care center. The crisis team decided to close all schools and day care centers in the city and district until June 12, 2020. Furthermore, all people at an accommodation center for asylum seekers and an elementary school in the district were required to be tested. One further case was identified in the school.

#### Outbreak 2

The two index cases of outbreak 2 were detected during routine SARS-CoV-2 testing prior to medical procedures in the UMG, according to the UMG test strategy. Following the positive test results of all household contacts of both index cases, immediate compulsory testing of all residents from RC2 was installed. All residents received face masks and written information material on COVID-19 in German and Romanian as, according to the LPHA, the proportion of Romanian-speaking residents was high (exact figures not available) (11, 12). The residents were required to attend testing in front of the building (supported by language mediation). After testing revealed positive results among 120 people, all residents in RC2 were quarantined between June 18 and 25, 2020 in order to organize contact tracing and to prevent transmission outside the building (11). For this purpose, the building was fenced off and access was monitored by security staff and police. Residents were informed about the measures by leaflets and mobile text messages, as well as oral information with translators.

The property management was required to present an IPC plan for RC2. Between June 20 and 21, 2020, a second test was offered to all people who had previously tested negative, in order for them to leave quarantine if their second result was negative after June 25, 2020. During quarantine, residents were supplied with groceries, meals and sanitary products. Opioid substitution treatment (OST) was provided to OST patients among residents, a mobile medical care center including psychiatric care and an information booth with Romanian language translation. Staff from community safety, security services and fire department, various emergency services, a low threshold addiction service, and the police service were involved in the operation. Items that were not provided by the public authorities were provided by local NGOs, such as baby food, diapers and telephone cards.

**Figure 3:**
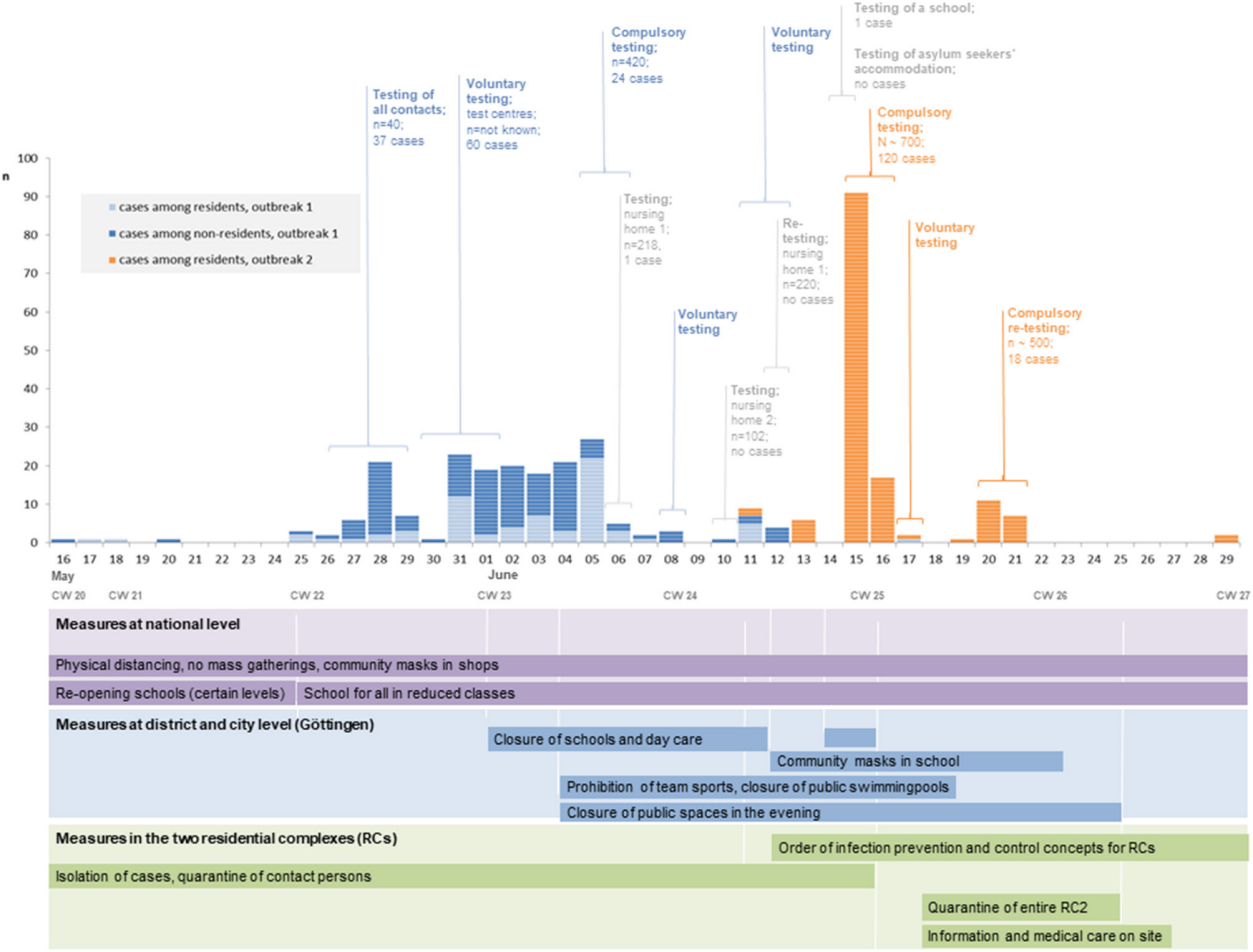
Distribution of SARS-CoV-2 cases in two residential complexes in Göttingen, May–June 2020 by date of testing and residential status of cases (COVID-19 database GA Göttingen, as of 03.07.2020) and implementation of control measures at national level, Göttingen county district and city level and in the two residential complexes, May–June 2020.

Further measures that were implemented at district level, e.g., a requirement to wear a mask on school premises (13), team sports were prohibited (14), and the closure of a public place in Göttingen city and others can be found in Figure 3.

## Discussion

The described outbreaks in RCs posed a particular challenge for both the city of Göttingen, due to high case numbers, as well as for the residents of the buildings, who were disproportionally affected by the imposed control measures. These outbreaks occurred when the first wave of the COVID-19 pandemic in Germany was over and most other districts succeeded in bringing down case numbers.

The high attack rates of 12 and 22% among residents of RC1 and RC2, respectively, suggest that, in line with published evidence (1–3), overcrowding, and within-household transmission have likely played a significant role in the spread of the virus. It was assumed by local authorities that in addition to officially registered residents further not-registered persons lived in the RCs, in particular in RC2, therefore overcrowding was likely even more severe. Physical distancing, self-isolation, and shielding may be difficult to implement in these settings (2, 15, 16). The high genomic sequence similarity of a subset of samples from outbreak 2 supports the hypothesis that all cases belonged to the same outbreak and there is no evidence of multiple introductions. Additionally, this is supported by phylogenetic analysis that revealed identical virus lineages B.1.159 in all samples. Despite relatively low overall lineage detection in Germany in 2020, B.1.159 was detected in only 9 other samples in Germany. However, the 19 analyzed cases were spread over the whole RC with at least one sequenced case per level in one of the four building parts, which supports our hypothesis of a fast transmission inside the building due to many contacts among residents.

At the time of the outbreaks, Lower Saxony was preparing to pass a Housing Protection Act, which allows municipalities to identify and declare overcrowded, run-down dwellings as uninhabitable. The act further defines a minimum space of 10 m^2^ per person. This could help prevent similar outbreaks which are associated with overcrowding.

The immediate compulsory testing of all residents and the quarantine of the entire building in outbreak 2 was initiated in response to the rapid spread of infection and low uptake of voluntary testing during outbreak 1. While it facilitated infection control measures, the crowding of people in front of the test center while waiting may have favored the further spread of the virus among residents. In the follow-up tests of those who initially had negative test results, 18 additional residents tested positive. These cases could be secondary cases. However, to exclude additional transmission during testing and quarantine/isolation, time scheduling and measures favoring physical distancing should therefore be considered when mass testing is implemented. Distribution of free masks can help to protect people in corridors and shared spaces.

To reduce the possibility of transmission within overcrowded buildings, it is recommended that cases, contact persons, and non-cases within narrow RCs are separated, as well as cases and negative-tested people within one household (15). The latter should be decided on a case-by-case basis with the aim to protect persons with risk of severe infection, and together with the household members (17). As the example of RC1 shows, separation can even be self-organized by residents within the building, if circumstances allow. However, this would not have been possible in the case of RC2, with much smaller apartments, lack of space and rooms, and more overcrowding. Therefore, municipalities should provide extra room for separation, such as in hostels or hotels.

Screening in workplaces of cases and contacts—as part of the Göttingen district test strategy—showed that the outbreak neither affected the two nursing homes where contact persons were employed, nor the schools of the infected children. However, even though the city implemented far-reaching measures in the general population to limit the outbreak, such as the closure of schools, day care centers, a public place and team sports (12, 14), overall case numbers in the whole district increased during the time of outbreak 1 and 2, and some of them were probably additionally related to the outbreaks. This is supported by the fact that total COVID-19 case numbers in the whole district surpassed cases assigned to the outbreaks during the outbreaks' period by 158 cases, of which 57 cases were assigned to a separate outbreak in the district, but decreased to very low levels below 10 reported cases per week after end of June (Figure 1). Contact tracing may have been hindered due to language barriers and high case numbers, which may have led to under-reporting.

Several factors might explain the overall low proportion (45%) of symptomatic COVID-19 cases in comparison to the national average of 85%. First, under-reporting of symptoms is likely due to the short period of time in which the interviews took place, as well as reported language barriers, particularly in RC2, where only 11% of the cases reported symptoms. The testing of all residents led to the identification of asymptomatic and presymptomatic cases, which was expected. Published data of different settings reveals that overall 40-45%, but with great variation and up to 96% of those infected with SARS-CoV-2 will remain asymptomatic, in particular among the young (18, 19). The infected population was comparatively young, with a median age of 23 and 20 years, respectively, and included a high proportion of cases among children under the age of 18 years. This is due to an overall young resident population in the two affected RC. We assume that the children played a major role in the outbreak, by playing together and transmitting the infection from household to household. Maybe the small elderly resident population was rather isolated and did not mix up with the families living in the residential complex, resulting in a lower AR of 5.9% and even 0 among persons aged 65 and older in RC1 and 2, respectively. The age distribution of residents and cases also explains the low proportion of hospitalized cases in both outbreaks of 4.8% compared to the national average of 17% in the same period (20). Nevertheless, severe cases requiring intensive medical care among people under 30 years and one death were also reported.

An important consideration of the two outbreaks and how they were handled are the experiences of the residents due to their social statuses and ethnic and cultural identities. As with other similar communities, many residents of the buildings may likely experience marginalization, discrimination, and racism (21–24). Discrimination and poverty are linked to precarious housing, living and working conditions; as well as to poor health conditions, including chronic underlying diseases that favor severe disease progression (25–32). Reducing the risk of infection in people living in overcrowded and precarious housing conditions is therefore important (31, 33). Particular emphasis should be placed on information about risk factors, identifying and close follow-up of people at increased risk of severe COVID-19 disease, and early initiation of inpatient care, if necessary. Overcrowded housing is a risk for pathogen transmission. Hence, the prevention of such housing situation has to be a task of public health.

Mass quarantine in the second outbreak was a substantial control measure that the city of Göttingen implemented due to the disproportionately high incidence already ranging between 71.5–89.9/100,000 population after the first outbreak, while the national incidence had come down to 2.6/100,000 at that time (5). During outbreak 2, the 7-day incidence in the city of Göttingen increased even to 123.5/100,000 population.

Residents and civil society organizations initially reacted with anger and protest to this measure: despite information flyers in German and Romanian language, and Romanian language translation, many residents of RC2 felt not well-informed about the necessity of the implemented measures. Insecurities arose from lack of information probably due to inappropriate or insufficient information as well as from the fear of infection inside the building, since physical distancing and self-isolation were hardly possible. To reach the entire resident population an even broader range of modes of information would have been helpful. Aspects like preferred information ways and lack of knowledge, distrust of authorities, or misinformation have to be taken into account. Furthermore, the consequences of an immediate restriction of movement for people who cannot store food, lack social support or are dependent on drugs and alcohol can be essential. The city supplied RC2 residents therefore with food, sanitary products, and provided OST as well as medical care. A quarantine may also jeopardize employment relationships, especially when they are informal. Therefore, quarantine should only be applied to direct contacts of cases. The local authorities quickly re-tested all negatively tested non-household contacts and released those who tested negative from the quarantine that is usually enacted for 14 days.

Populations that are deemed “marginalized” have been disproportionately affected by the COVID-19 pandemic and coercive public health measures (16, 24, 34). Differing proficiencies in the locally spoken language of affected people might also play a role. The economical and psychological consequences and effects of measures vary according to the socio-economic situation of the people affected (28, 35).

Of note is the higher AR among women in RC2 as compared to men. We can only speculate reasons for that: women may have been more exposed to infected, mostly asymptomatic children in the household. It is crucial to adequately identify the most vulnerable in the outbreak situation, and to consider specific needs by involving community members or representative organizations of the residents, such as NGOs, key people in the community, language mediators, social workers, and counselors in the planning and implementation of measures (16, 36–38). The current COVID-19 pandemic as well as the HIV and Ebola epidemics have shown that the effectiveness of infection control measures depends largely on community participation (37–40). In the case of Göttingen, some requested NGOs refused their support because they did not want to enter the building or were not available at short notice. Preventive networking is therefore essential to be prepared when it comes to an outbreak. An anti-discriminatory approach, transparent communication, and information in all needed languages and consideration of actual needs can facilitate voluntary testing and individual quarantine and isolation (17).

Lastly, a lesson that was learned also in Göttingen, ethnization and culturalization of social problems should be avoided because they reproduce discrimination and prejudices. It is particularly important to ensure that COVID-19 is not associated with certain communities (27, 33). Ethnic groups and affiliation to religious or social communities should only be named if relevant for outbreak management. In general, public health measures should be implemented in a just and equitable manner (24).

There are several limitations to our analysis: The presentation of the time course of the outbreaks was significantly influenced by the screening activities of the LPHA, and the actual course of the outbreaks is not known. We may have missed further cases. The data used for descriptive analyses was derived from the LPHA's COVID-19-database as of Jul 3, 2020. Any information that may have subsequently been added on cases is not included in the present analysis.

In these outbreaks the determination of the secondary or tertiary attack rate due to transmission within households or inside the buildings would have been important, however, this could not be done since the available data did not provide any information on whether a person was a primary, secondary, or tertiary case. The screenings were not done systematically as not all residents showed up for all screenings. Thus, residents who were tested positive in the last screening might have already been infected earlier. Furthermore, information on onset of symptoms was largely incomplete in the epidemiological dataset so that this information could also not be used.

Not all officially registered residents of the affected RCs lived there at the time of the outbreaks, and there might be residents living in the RCs who were not officially registered. Inaccuracies are therefore to be expected in the number and description of the socio-demographics of the residents. Furthermore, only cumulative information on age and gender of residents was available for analysis so that further analyses of cases and residents were not possible. We had no information on jobs, time spent at home or outside, number of persons per household neither of the infected nor of the resident population, so that further analyses on household level were not possible.

Finally, a total of 333 cases were assigned to the outbreaks in the LPHA's COVID-19 database, but only 287 cases were assigned to this outbreak in SurvNet. This difference is caused by incomplete assignment of reported cases to outbreaks in SurvNet, which may be due to the fact that the Göttingen LPHA uses different software products: SurvNet is only used to transmit case reports to the federal and national level, but not as working software for documentation, which may cause a loss of important data.

## Conclusion

In outbreaks involving people who are affected by poverty and precarious working and living conditions, special sensitivity in all implemented measures is recommended. Discrimination and racism should be acknowledged as crucial determinants of health disparities. The heterogeneity and specific needs of residents should be taken into account, and language- and culturally-sensitive information of residents about all measures as well as a participatory approach are recommended.

The findings of the two outbreaks in Göttingen indicate that overcrowded housing conditions can promote COVID-19 outbreaks. Effective public health measures included the immediate PCR testing of all residents after the occurrence of initial cases, implementation of hygiene measures in the buildings, and the general closure of schools and hygiene measures at local level. Further recommended measures include the implementation of a legal basis to declare buildings with cramped housing as uninhabitable to prevent unhealthy living, and organizing spatial separation possibilities.

## Data Availability Statement

Aggregated data from a limited version of the German surveillance system database can be retrieved via SurvStat@RKI 2.0; https://survstat.rki.de/. Detailed data are confidential and protected by German law and are available from the corresponding author upon reasonable request. The data generated from genomic sequencing was deposited and made publicly available in ENA via https://www.ebi.ac.uk/ena/browser/view/PRJEB45860.

## Ethics Statement

Ethical review and approval or specific consent procedures were not required for this study in accordance with the local legislation and institutional requirements. The outbreak investigation presented in this manuscript was conducted as part of the official tasks of the local public health authorities of the respective district, supported by the Robert Koch Institute (RKI) as the national Public Health Institute upon official request in accordance to §4 of the German Protection against Infection Act. Therefore, this investigation was exempt from additional institutional review.

## Author Contributions

RZ and NS drafted the manuscript and as members of the outbreak investigation team from the RKI conceptualized the analyses, analyzed, and interpreted the data with support of KAl, VB, TE, SK, and UR. KAl, VB, TE, and UR supervised and were responsible for epidemiological field visits during the COVID-19 pandemic. DT-T and AP collected the data and, together with EM, KAz, PB, and SS planned and implemented local infection control measures in the city of Göttingen. They were supported by NF and TA. MH and SK were responsible for whole genome sequencing analyses and interpretation of the results. All authors critically revised the manuscript and approved the final version.

## Conflict of Interest

The authors declare that the research was conducted in the absence of any commercial or financial relationships that could be construed as a potential conflict of interest.

## Publisher's Note

All claims expressed in this article are solely those of the authors and do not necessarily represent those of their affiliated organizations, or those of the publisher, the editors and the reviewers. Any product that may be evaluated in this article, or claim that may be made by its manufacturer, is not guaranteed or endorsed by the publisher.

## Supplementary Material

Supplementary Figure 1 Location plan of RC2. Each grey square represents one sample that was whole genome sequenced (*n* = 19). Samples were selected considering multiple factors to cover the outbreak situation, such as location of the flats of the cases within RC2, cases from different age groups.

Supplementary Figure 2 Illustration of high sequence similarity between the outbreak samples. A multiple sequence alignment summarizing the genetic variation for samples which were whole genome sequenced. The top panel shows an overview of the whole SARS-CoV-2 genome of all 19 samples, with variant positions for individual sequences indicated in a darker color and a yellow box highlighting broad regions where those variants are located. The bottom panel shows the actual nucleotide (or gap) present at each location in the genome which differed in at least one sequence. As show in the figure, only five positions show any variation in at least one of the 19 sequences.

## Abbreviations

RC: Residential Complex
RKI: Robert Koch Institute
LPHA: Local Public Health Authority
CW: calendar week
AR: attack rate
WGS: Whole genomes sequencing.
