# Supplementary figures and images for: COVID-19 Outbreaks in Settings With Precarious Housing Conditions in Germany: Challenges and Lessons Learned
Source: Front Public Health. 2021 Sep 21;9:708694. doi: 10.3389/fpubh.2021.708694 (PMC8490676; doi:10.3389/fpubh.2021.708694)

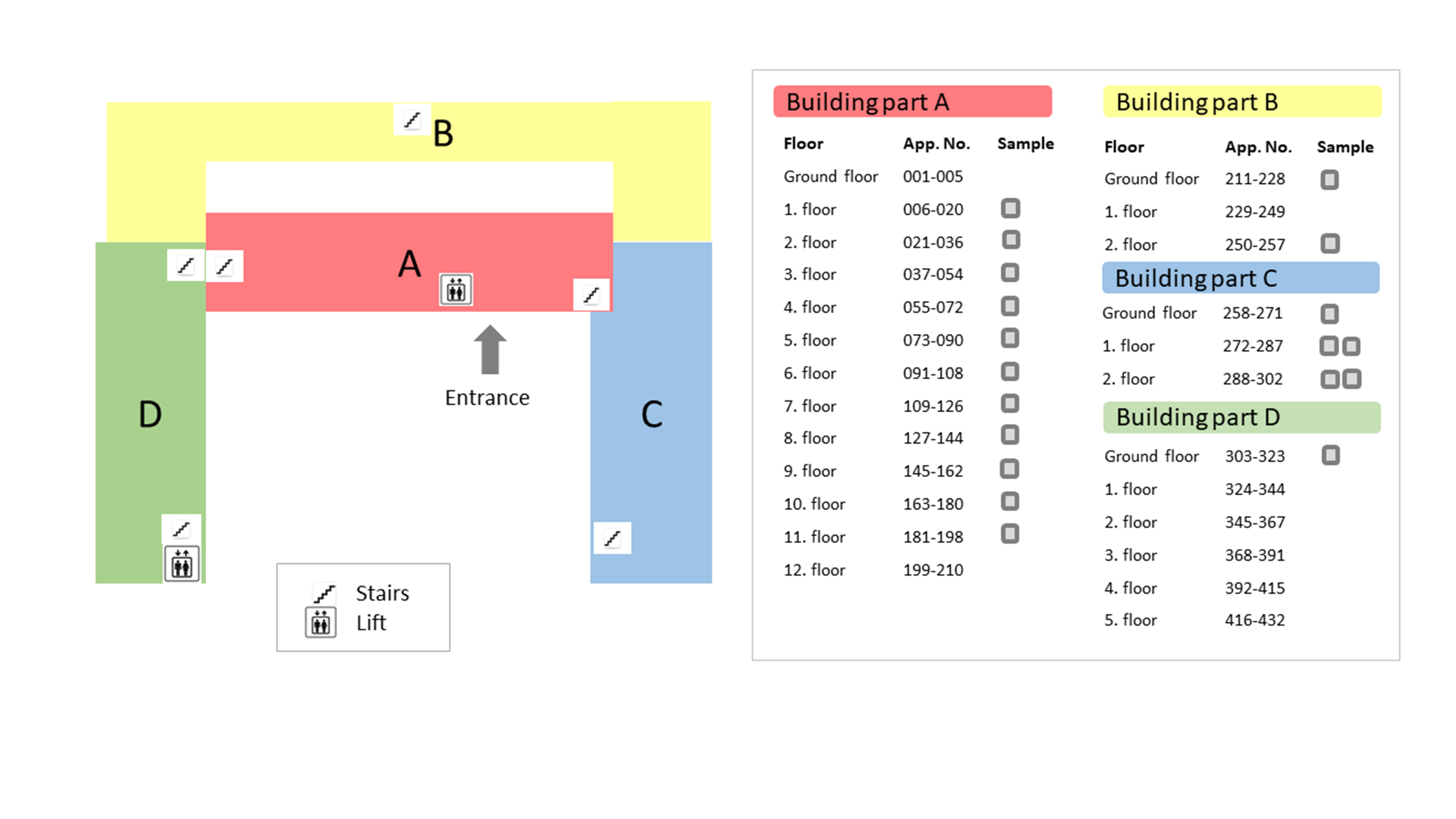

Supplement: Supplementary Figure 1 — Location plan of RC2. Each grey square represents one sample that was whole genome sequenced (n = 19). Samples were selected considering multiple factors to cover the outbreak situation, such as location of the flats of the cases within RC2, cases from different age groups. [file Image_1.TIF]

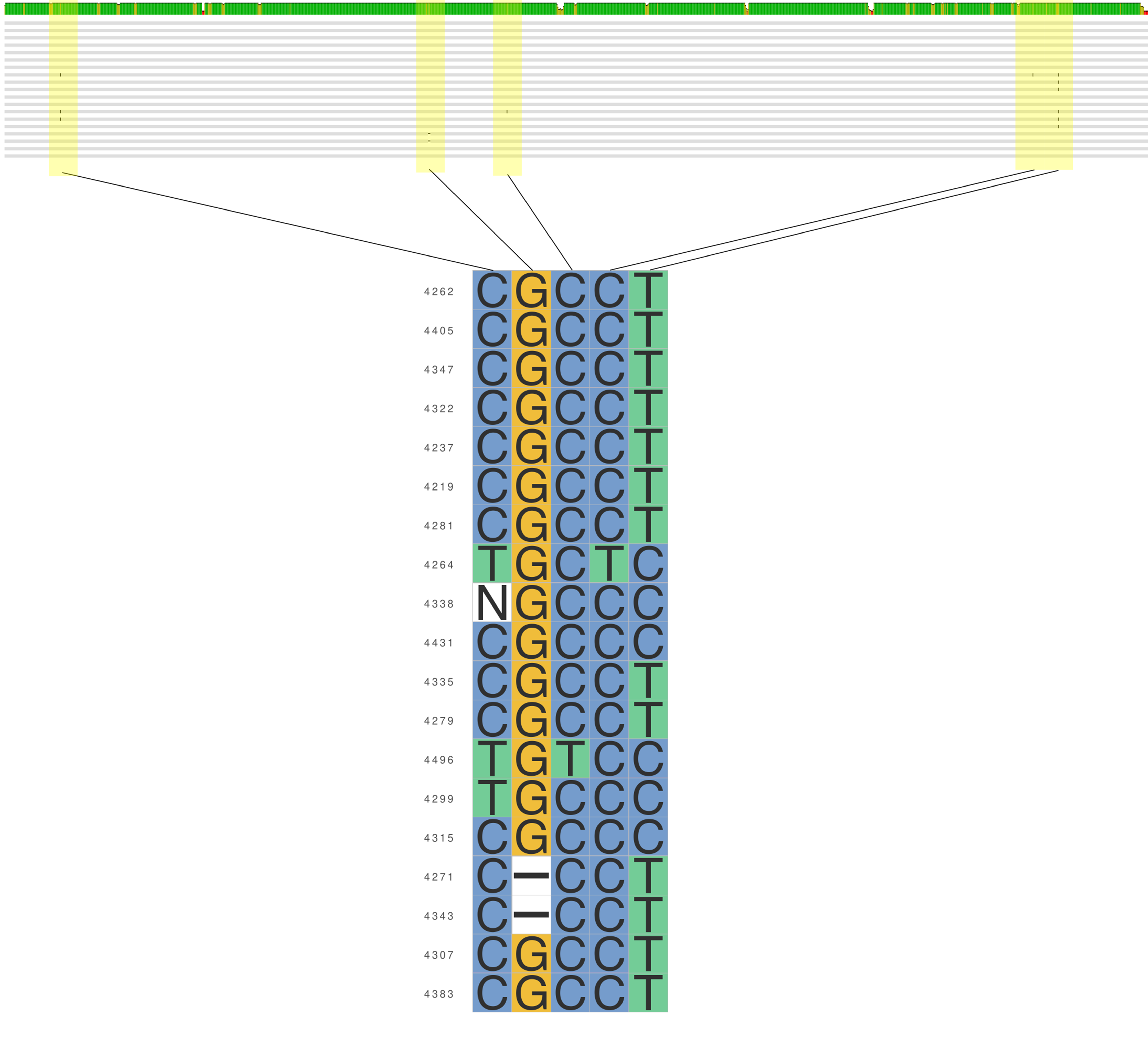

Supplement: Supplementary Figure 2 — Illustration of high sequence similarity between the outbreak samples. A multiple sequence alignment summarizing the genetic variation for samples which were whole genome sequenced. The top panel shows an overview of the whole SARS-CoV-2 genome of all 19 samples, with variant positions for individual sequences indicated in a darker color and a yellow box highlighting broad regions where those variants are located. The bottom panel shows the actual nucleotide (or gap) present at each location in the genome which differed in at least one sequence. As show in the figure, only five positions show any variation in at least one of the 19 sequences. [file Image_2.PNG]
